# Using re-randomization to increase the recruitment rate in clinical trials – an assessment of three clinical areas

**DOI:** 10.1186/s13063-016-1736-z

**Published:** 2016-12-13

**Authors:** Brennan C. Kahan

**Affiliations:** Pragmatic Clinical Trials Unit, Queen Mary University of London, 58 Turner St, London, E1 2AB UK

**Keywords:** Re-randomization, Randomized controlled trials, Recruitment, Efficient trial design

## Abstract

**Background:**

Patient recruitment in clinical trials is often challenging, and as a result, many trials are stopped early due to insufficient recruitment. The re-randomization design allows patients to be re-enrolled and re-randomized for each new treatment episode that they experience. Because it allows multiple enrollments for each patient, this design has been proposed as a way to increase the recruitment rate in clinical trials. However, it is unknown to what extent recruitment could be increased in practice.

**Methods:**

We modelled the expected recruitment rate for parallel-group and re-randomization trials in different settings based on estimates from real trials and datasets. We considered three clinical areas: in vitro fertilization, severe asthma exacerbations, and acute sickle cell pain crises. We compared the two designs in terms of the expected time to complete recruitment, and the sample size recruited over a fixed recruitment period.

**Results:**

Across the different scenarios we considered, we estimated that re-randomization could reduce the expected time to complete recruitment by between 4 and 22 months (relative reductions of 19% and 45%), or increase the sample size recruited over a fixed recruitment period by between 29% and 171%. Re-randomization can increase recruitment most for trials with a short follow-up period, a long trial recruitment duration, and patients with high rates of treatment episodes.

**Conclusions:**

Re-randomization has the potential to increase the recruitment rate in certain settings, and could lead to quicker and more efficient trials in these scenarios.

## Background

Recruitment to randomized controlled trials (RCTs) is challenging, and many trials are either stopped early due to insufficient recruitment, or require longer to complete than expected [1–5]. This can have adverse impacts on patient care. Trials which are terminated early will be underpowered, and may not produce high-quality evidence. Poor recruitment can make conducting trials in certain areas infeasible; this can be particularly problematic for trials in rare diseases [6, 7]. Poor recruitment can also lead to concerns regarding the ethics of exposing participants to the potential harms of taking part in the trial and then failing to use their contribution if recruitment proves infeasible. Longer recruitment periods are also problematic, as they lead to higher costs, allowing fewer trials overall to be funded. Furthermore, longer recruitment periods will delay trial results being known, leading to delays in successful interventions being adopted into routine care, or in unsuccessful or harmful treatments being discontinued. Poor recruitment is a major barrier to conducting effective RCTs, and has been identified as the top research priority for leads of UK Clinical Trials Units [8].

The re-randomization design has been proposed as a way of increasing the recruitment rate compared to parallel-group trials [9]. The re-randomization design involves re-enrolling and re-randomizing patients who require further treatment after their initial enrollment is complete. An overview of this design is provided in Table 1, and further details are available in a previous publication [9]. The re-randomization design can be used in situations where (1) patients may require treatment on multiple occasions, (2) the intervention(s) under study would be used for each new treatment episode, and (3) the intervention duration and length of the follow-up period for each randomization are less than the overall length of the trial recruitment period.

**Table 1:** Overview of re-randomization trials

Setting requirements for re-randomization trials	1) Some patients may require treatment on multiple occasions 2) The intervention(s) would be used for each new treatment episode 3) The intervention duration and length of the follow-up period for each treatment episode are less than the overall length of the trial recruitment period
Design requirements for re-randomization trials	1) Patients are only re-enrolled and re-randomized when they have completed the follow-up period from their previous randomization 2) Randomizations for the same patient are performed independently
Implementation of re-randomization trials	1) Patients are enrolled as usual, randomized to a treatment group, and followed-up until all outcomes have been collected 2) If patients experience new treatment episodes and require further treatment, they can be re-enrolled and re-randomized, provided they have completed the follow-up period from their previous randomization 3) This process is repeated until the target sample size is met

Re-randomization trials can provide unbiased estimates of treatment effect and correct type I error rates provided: (1) patients are only re-enrolled and re-randomized after the follow-up period from their previous treatment episode is complete, and (2) randomizations for the same patient are performed independently [9]. It is important to note that the number of times that each patient is enrolled is not specified in advance, and instead depends on the number of treatment episodes that they experience during the course of the trial. For example, some patients may be enrolled once; others may be enrolled multiple times.

Then, under the assumption that the treatment effect is constant (i.e. that the intervention confers the same benefit relative to control across all patients and all treatment episodes), and that the variance of the outcome is the same under re-randomization as it would be in a parallel-group design (i.e. that the variance is not increased in a re-randomization trial), re-randomization will have the same power as a parallel-group trial with an equivalent number of observations [9]. This result is independent of intraclass correlation coefficient (i.e. the degree of correlation between treatment episodes from the same patient); full details are available in a previous publication [9].

This implies that when the above assumptions are reasonable, the same sample size calculation as in a parallel-group design could be used, but instead of recruiting the specified number of patients, a re-randomization trial could recruit the specified number of treatment episodes. For example, instead of recruiting 200 individual patients, the re-randomization design would recruit 200 treatment episodes. Because some patients will contribute multiple treatment episodes, this will increase the recruitment rate compared to a parallel-group trial, thereby allowing re-randomization trials to be conducted more quickly. In scenarios where the above assumptions are not likely, re-randomization could still be used, provided it meets the criteria outlined in Table 1. However, a larger number of treatment episodes may be required, although in some cases this would still require fewer overall patients than a parallel-group design, and so would still facilitate quicker recruitment.

Despite the potential advantages of the re-randomization design in terms of increased recruitment rate, there has been little previous research on its potential impact, and it is, therefore, unknown how much of an effect on recruitment it might have in practice. We therefore conducted a modelling study to assess the impact that re-randomization could have on recruitment in three different clinical areas: (1) in vitro fertilization (IVF), (2) severe asthma exacerbations, and (3) acute sickle cell pain crises.

## Methods

We begin by examining some of the factors that will influence how much of an advantage re-randomization can provide. Over a fixed recruitment duration, the increased recruitment from a re-randomization trial versus a parallel-group trial can be measured by:

$$ \frac{Number\  enrolled\  treatment\  episodes}{Number\  enrolled\  patients} $$

i.e. it measures the number of extra treatment episodes that we would recruit through re-randomization as compared to a parallel-group design.

The number of extra enrolled treatment episodes is determined by (1) the total number of treatment episodes that occur during the trial recruitment period and (2) the proportion of these treatment episodes that are enrolled in the trial.

Some of the key factors that can affect (1) and (2) above (and thus determine the recruitment benefit conferred through re-randomization) are:

- *The rate of treatment episodes*: higher rates lead to higher numbers of treatment episodes
- *The length of the recruitment period*: longer recruitment periods will lead to higher numbers of treatment episodes
- *The recruitment trajectory of new patients:* if most new patients are recruited towards the end of the trial, this could lead to lower numbers of treatment episodes compared to a constant recruitment trajectory (as there would be less time for patients recruited towards the end of the trial to experience new treatment episodes)
- *The length of the follow-up period*: because we cannot enroll patients until the follow-up period from their previous enrollment is complete, shorter follow-up periods will lead to larger proportions of treatment episodes that can be enrolled in the trial
- *Limits on the number of enrollments per patient*: lower limits will lead to lower proportions of treatment episodes that are enrolled
- *The probability of patient’s re-consenting for re-enrollment:* lower numbers of patients who re-consent to re-enroll for their 2nd, 3rd, 4th, etc., treatment episode will lead to a lower proportion of treatment episodes that are enrolled

From this, we can see that re-randomization trials will increase recruitment the most when there is a short follow-up period, a long trial recruitment period, and patients have a high rate of treatment episodes. Instituting small limits on the number of enrollments allowed per patient will reduce the benefit from re-randomization, as will low rates of re-consent from patients for subsequent treatment episodes.

### Modelling study

We modelled the expected recruitment rates for parallel-group and re-randomization designs across three clinical areas. For each clinical area, we considered several scenarios based on different sample size targets and different recruitment durations for a parallel-group design. We considered both small and large sample size targets and short and long recruitment durations. This led to four unique scenarios for each clinical area: (1) small sample size, short recruitment duration, (2) small sample size, long recruitment duration, (3) large sample size, short recruitment duration, and (4) large sample size, long recruitment duration.

Within each of the three clinical scenarios, we selected two published or ongoing trials on which to base the sample size targets and recruitment durations (exact values listed below). There was no formal or systematic process for identifying or selecting potential trials; instead, we considered any trials we were aware of, provided they would have been appropriate for a re-randomization design. We selected two trials for each clinical area on the basis that the trials provided diverse sample sizes and recruitment durations.

To model recruitment from a parallel-group trial, we assumed a constant recruitment rate throughout the recruitment period. We calculated the monthly recruitment rate by dividing the target sample size by the total recruitment duration (in months).

To model the recruitment rate for re-randomization trials, we assumed that the same number of new patients (not previously enrolled in the trial) would be recruited each month as in a parallel-group design. We therefore used the same monthly recruitment rate for new patients as for a parallel-group trial. We then assumed that a certain proportion of newly recruited patients would be re-randomized during the remainder of the recruitment period. These proportions were estimated from published studies, and are listed below. We estimated the total recruitment in each month for re-randomization trials by adding the number of newly enrolled patients and the number of re-randomized patients together.

We then estimated the cumulative recruitment for both parallel-group and re-randomization trials. We compared recruitment between the two different designs in two ways; (1) the time to complete recruitment and (2) the sample size recruited over a fixed time period. We calculated the time to complete recruitment as the month in which the cumulative recruitment passed the sample size target (note that that the time to complete recruitment for parallel-group designs was set to be equal to the recruitment duration discussed earlier). We calculated the sample size recruited over a fixed time period as the cumulative recruitment at the end of the specified recruitment duration for the parallel-group trial (i.e. we estimated what the cumulative recruitment for a re-randomization trial would have been if it had continued to recruit over the same time frame as the parallel-group trial).

For re-randomization trials, we instituted limits on the number of times that each patient could be enrolled [9]. We used two limits: (1) a smaller number of enrollments and (2) a larger number of enrollments. The exact values we used for the limits are listed below.

We provide further details on the assumptions made for each of the three clinical areas below.

### In vitro fertilization

IVF is a technique to help people or couples with fertility problems become pregnant. Some trials comparing different methods of IVF may be suitable for re-randomization (Table 2), because: (1) some participants who do not become pregnant after their first IVF cycle may undergo further cycles [10], (2) some interventions in this area are designed to be used for each new cycle, and (3) the duration of these interventions is often short-term (e.g. during the cycle), and follow-up is often complete once it is determined that a cycle was unsuccessful.

**Table 2:** Suitability of re-randomization in the setting of (1) in vitro fertilization, (2) severe asthma exacerbations, and (3) acute sickle cell pain crises

Clinical area	Setting requirement	Justification
In vitro fertilization	1) Some patients may require treatment on multiple occasions	Some participants who do not become pregnant after their first IVF cycle may undergo further cycles
	2) The intervention(s) would be used for each new treatment episode	The interventions considered (minimal stimulation, sperm selection) are designed to be used for each new cycle
	3) The intervention duration and length of the follow-up period for each treatment episode are less than the overall length of the trial recruitment period	The interventions considered (minimal stimulation, sperm selection) are short-term (during the IVF cycle) The follow-up period will vary between trials, but will often be complete once it is determined a cycle was unsuccessful
Severe asthma exacerbations	1) Some patients may require treatment on multiple occasions	Some patients may experience more than one exacerbation during a given time period
	2) The intervention(s) would be used for each new treatment episode	The interventions considered (magnesium sulphate) are designed to be used for each new exacerbation
	3) The intervention duration and length of the follow-up period for each treatment episode are less than the overall length of the trial recruitment period	The interventions considered (magnesium sulphate) is short-term, and given while a patient is experiencing an exacerbation. The follow-up period will vary between trials, but is often relatively short-term
Acute sickle cell pain crises	1) Some patients may require treatment on multiple occasions	Some patients may experience more than one pain crisis during a given time period
	2) The intervention(s) would be used for each new treatment episode	The interventions considered (nitric oxide gas, ketoprofen) are designed to be used for each new pain crisis
	3) The intervention duration and length of the follow-up period for each treatment episode are less than the overall length of the trial recruitment period	The interventions considered (nitric oxide gas, ketoprofen) is short-term, and given while a patient is experiencing a pain crisis. The follow-up period will vary between trials, but is often relatively short-term

We chose two trials as the basis for our modelling study. The first trial compared minimal stimulation in IVF to standard IVF [11]. The sample size was 564 participants, and the recruitment duration was 55 months. The second trial compared a new method of sperm selection to the standard method (http://www.habselect.org.uk/). The trial is not yet complete; the anticipated sample size is 3730 couples, and the anticipated recruitment duration is 21 months. Based on these two trials, we used small and large sample sizes of 564 and 3730, respectively, and short and long recruitment durations of 21 and 55 months, respectively.

We note that because the second trial is not yet complete, the final sample size and/or the recruitment duration may differ to what we have assumed here; if the true recruitment duration is longer than that assumed here, then our results will underestimate the benefit of re-randomization (and vice versa).

For re-randomization trials we set the smaller and larger limit on the number of enrollments for each patient as two and three IVF cycles, respectively. For a limit of two IVF cycles, we estimated that 60% of participants would undergo one cycle that and 40% would undergo two [12]. For a limit of three IVF cycles, we estimated that 60% of participants would undergo one cycle, 25% would undergo two cycles and that 15% would undergo three cycles [12]. We set a 6-month waiting period between IVF cycles, as this is recommending by some clinical commissioning groups in the UK. We note that this policy may not be typical for all trials, and as such, our results may underestimate the benefit of re-randomization.

We calculated the number of re-randomized treatment episodes each month using the following steps. First we modelled the number new enrollments each month using the same approach as for parallel-group trials. We then calculated the expected number of IVF cycles that would occur each month for patients who had previously been enrolled (accounting for the limit on the number of times each patient was allowed to be enrolled). The monthly recruitment rate under re-randomization was then calculated by adding the number of new enrollments each month to the number of re-randomizations that occurred each month. Further details on these calculations are available in the Appendix.

### Severe asthma exacerbations

People with asthma sometimes experience severe asthma exacerbations which may require treatment in hospital. Some trials comparing different methods of treating severe asthma exacerbations may be suitable for re-randomization (Table 2), because: (1) some people may experience more than one exacerbation during a given time period [13], (2) most interventions to treat exacerbations are designed to be used for each new exacerbation, and (3) interventions are often short-term (e.g. while the patient is experiencing an exacerbation), and length of follow-up is often relatively short-term.

We chose two trials as the basis for our modelling study. The first trial compared intravenous and nebulized magnesium sulphate with placebo [14]. The target sample size was 1200; however, due to slower than anticipated recruitment the trial stopped early after 1109 patients were enrolled. The recruitment duration was 47 months. The second trial also compared nebulized magnesium sulphate with placebo [15]. The sample size was 508, and the recruitment duration was 27 months. Based on these two trials, we used small and large sample sizes of 508 and 1200, respectively, and short and long recruitment durations of 27 months and 47 months, respectively.

For re-randomization trials we set the smaller and larger limit on the number of enrollments for each patient as two and four treatment episodes, respectively. We also assumed a 1-month follow-up period, meaning that patients could not be re-randomized for at least 1 month after their previous enrollment. We estimated the exacerbation rate as 0.52/6 months [13], equating to a monthly rate of 0.087. We assumed that this rate followed a Poisson distribution.

We calculated the number of re-randomized treatment episodes each month using the following steps. First we modelled the number new enrollments each month using the same approach as for parallel-group trials. We then calculated the expected number of exacerbations that newly enrolled patients would have between the end of their follow-up period and the end of the recruitment period (accounting for the limit on the total number of times each patient was allowed to be enrolled). We assumed that on average these exacerbations would occur uniformly across the remaining months, and that patients enrolled in the trial once would be enrolled again for all subsequent exacerbations. The monthly recruitment rate under re-randomization was then calculated by adding the number of new enrollments each month to the number of re-randomizations that occurred each month. Further details on these calculations are available in the Appendix.

### Acute sickle cell pain crises

People with sickle cell disease sometimes experience painful sickle cell crises, which often require hospitalization for treatment. Some trials comparing different methods of treating acute sickle cell pain crises may be suitable for re-randomization (Table 2), because: (1) some people may experience more than one pain crisis during a given time period [16], (2) most interventions to treat pain crises are designed to be used for each new pain crisis, and (3) interventions are often short-term (e.g. while the patient is experiencing a pain crisis) and length of follow-up is often relatively short-term.

We chose two trials as the basis for our modelling study. The first trial compared inhaled nitric oxide gas with placebo [17]. The sample size was 150 and the recruitment duration was 49 months. The second trial compared ketoprofen with placebo [18]. The sample size was 66 and the recruitment duration was 32 months. Based on these two trials, we used small and large sample sizes of 66 and 150, respectively, and short and long recruitment durations of 32 months and 49 months, respectively.

For re-randomization trials we set the smaller and larger limit on the number of enrollments for each patient as two and four treatment episodes, respectively. We also assumed a 1-month follow-up period, meaning that patients could not be re-randomized for at least 1 month after their previous enrollment. The rate of hospitalizations for people with sickle cell disease is 1.5/year [16]; however, only 76.9% of hospitalizations were for pain crises. We therefore estimated the rate of hospitalizations for acute pain crises as 1.15/year, equating to a rate of 0.096/month. We assumed that this rate followed a Poisson distribution.

We calculated the number of re-randomized treatment episodes each month using the same approach as for severe asthma exacerbations; full details of these calculations can be found in the Appendix.

## Results

### In vitro fertilization

**Fig. 1:**
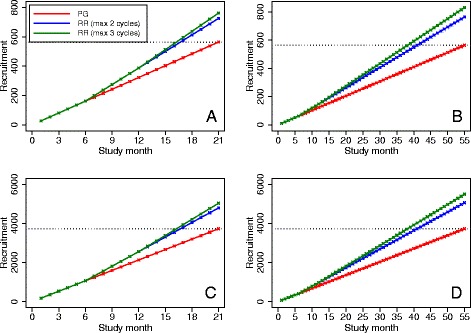
Comparison of recruitment rates between re-randomization and parallel-group designs in in vitro fertilization (IVF) trials. *PG parallel-group, *RR* re-randomization. The *red line* denotes recruitment for a parallel-group trial. The *blue line* denotes recruitment for a re-randomization trial where each participant may be enrolled for a maximum of two treatment cycles. The *green line* denotes recruitment for a re-randomization design where each participant may be enrolled for a maximum of three treatment cycles. The *dotted line* denotes the sample size target. For the re-randomization designs, treatment cycles were 6 months apart. Panel **a** denotes a small trial (*n* = 564) with a short recruitment duration (21 months). Panel **b** denotes a small trial (*n* = 564) with a long recruitment duration (55 months). Panel **c** denotes a large trial (*n* = 3730) with a short recruitment duration (21 months). Panel **d** denotes a large trial (*n* = 3730) with a long recruitment duration (55 months)

Results are shown in Fig. 1. Compared to a parallel-group design, re-randomization reduced the expected time to complete recruitment by between 4 months (17 versus 21 months; 19% relative reduction) and 17 months (38 versus 55 months; 31% relative reduction) across different scenarios. Over a fixed recruitment period, re-randomization increased the sample size by between 29% (726 versus 564) and 47% (831 versus 564).

### Severe asthma exacerbations

**Fig. 2:**
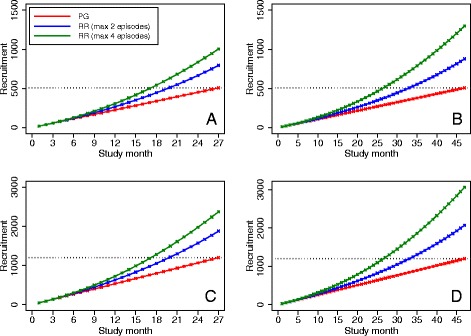
Comparison of recruitment rates between re-randomization and parallel-group designs in trials of asthma exacerbations. *PG parallel-group, *RR* re-randomization. The *red line* denotes recruitment for a parallel-group trial. The *blue line* denotes recruitment for a re-randomization trial where each participant may be enrolled for a maximum of two treatment episodes. The *green line* denotes recruitment for a re-randomization design where each participant may be enrolled for a maximum of four treatment episodes. The *dotted line* denotes the sample size target. Panel **a** denotes a small trial (*n* = 508) with a short recruitment duration (27 months). Panel **b** denotes a small trial (*n* = 508) with a long recruitment duration (47 months). Panel **c** denotes a large trial (*n* = 1200) with a short recruitment duration (27 months). Panel **d** denotes a large trial (*n* = 1200) with a long recruitment duration (47 months)

Results are shown in Fig. 2. Compared to a parallel-group design, re-randomization reduced the expected time to complete recruitment by between 7 months (20 versus 27 months; 26% relative reduction) and 20 months (27 versus 47 months; 43% relative reduction) across different scenarios. Over a fixed recruitment period, re-randomization increased the sample size by between 56% (795 versus 508) and 156% (1299 versus 508).

### Acute sickle cell pain crises

**Fig. 3:**
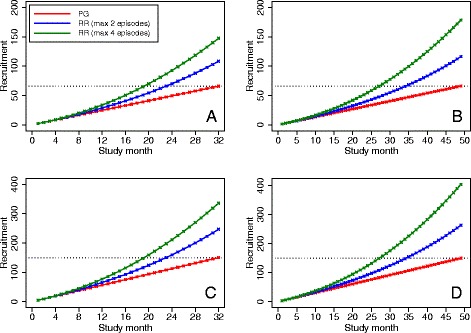
Comparison of recruitment rates between re-randomization and parallel-group designs in trials of acute sickle cell pain crises. *PG parallel-group, *RR* re-randomization. The *red line* denotes recruitment for a parallel-group trial. The *blue line* denotes recruitment for a re-randomization trial where each participant may be enrolled for a maximum of two treatment episodes. The *green line* denotes recruitment for a re-randomization design where each participant may be enrolled for a maximum of four treatment episodes. The *dotted line* denotes the sample size target. Panel **a** denotes a small trial (*n* = 66) with a short recruitment duration (32 months). Panel **b** denotes a small trial (*n* = 66) with a long recruitment duration (49 months). Panel **c** denotes a large trial (*n* = 150) with a short recruitment duration (32 months). Panel **d** denotes a large trial (*n* = 150) with a long recruitment duration (49 months)

Results are shown in Fig. 3. Compared to a parallel-group design, re-randomization reduced the expected time to complete recruitment by between 9 months (23 versus 32 months; 28% relative reduction) and 22 months (27 versus 49 months; 45% relative reduction) across different scenarios. Over a fixed recruitment period, re-randomization increased the sample size by between 64% (108 versus 66) and 171% (179 versus 66).

## Discussion

Poor recruitment is a common problem in RCTs, and is a major barrier to conducting effective trials. In this study we evaluated the potential impact of re-randomization on recruitment. We found that it can substantially increase the recruitment rate in some situations, allowing trials to be completed more quickly. At the extreme, we estimated that re-randomization could reduce the recruitment duration by almost 2 years for a trial of acute sickle cell pain crises (from 49 to 27 months, a 45% reduction). However, benefits were not always so large; in one scenario, re-randomization reduced the recruitment period by only four months (from 21 to 17 months, a 19% reduction). Alternatively, re-randomization could be used to increase the sample size compared to a parallel-group design; we estimated that over a fixed recruitment period, re-randomization increased the number of treatment episodes between 29% and 171%.

The potential benefits of re-randomization depend on several factors, including the rate of treatment episodes, the length of the follow-up period, and the duration of the trial recruitment period. Other factors, such as the recruitment trajectory for new patients, the probability that patients re-enroll for subsequent treatment episodes, and whether there are limits on the number of treatment episodes per patient will also affect the recruitment rate for re-randomization trials.

Our study had several limitations. We chose the modelling parameters based on real trials and datasets; however, we used only a limited number of examples. It is possible that using parameters based on other trials or datasets may have given different results. We also assumed a constant recruitment trajectory, and assumed that patients would re-consent for each new treatment episode during the trial period. These assumptions may not always be valid in practice, and if violated would lead to reduced recruitment from re-randomization. However, we generally opted to use more conservative parameter estimates when possible, which may have led to more conservative estimates of benefit. For example, we chose the rate of asthma exacerbations as 1.04/year [13]; however, other studies have reported rates of up to 3.2/year [19]. We also instituted limits on the number of randomizations per patient; however, we note that there is as of yet no consensus on whether limits should be routinely imposed in re-randomization trials; for trials that do not limit the number of randomizations, our results may therefore underestimate the benefit of re-randomization.

The re-randomization design is a relatively new proposal, and as such, much of the methodology research surrounding best practice for the design, conduct, and analysis of such trials is still ongoing. Further information on design and analysis issues for re-randomization trials are available elsewhere [9, 10, 20].

Although re-randomization can provide benefit in terms of recruitment, it should only be used in appropriate settings, such as when (1) patients may require treatment on multiple occasions, (2) the intervention(s) under study would be used for each new treatment episode, and (3) the intervention duration and length of the follow-up period for each randomization are less than the overall length of the trial recruitment period. Using re-randomization in other settings (e.g. for interventions which would be used only once in practice, or situations where re-randomization would have to occur before the follow-up period from the previous enrollment is complete) could lead to bias or inaccurate conclusions.

## Conclusion

Re-randomization has the potential to increase the recruitment rate in certain settings, and could lead to quicker and more efficient trials in these scenarios.

## Appendix

### Estimating recruitment under re-randomization

#### In vitro fertilization

We calculated the cumulative recruitment as follows. Let:

- *a*_*i*_ = the expected number of new enrollments in the *i* ^th^ month (where *a*_*i*_ is constant across all *i*)
- *b*_*i*_ = the expected number of re-randomizations in the *i* ^th^ month
- *CR*_*m*_ = the cumulative recruitment at the end of month *i* = *m*

Then, for a limit of two enrollments per participant, we calculated *b*_*i*_ as:

$$ {b}_i=0.4{a}_{i-6}. $$

For a limit of three enrollments per participant, we calculated *b*_*i*_ as:

$$ {b}_i=0.4{a}_{i-6}+0.15{a}_{i-12}. $$

Finally, we calculated *CR*_*m*_ as:

$$ C{R}_m={\displaystyle \sum_{i=1}^m}\left({a}_i+{b}_i\right). $$

#### Severe asthma exacerbations

We calculated the cumulative recruitment as follows. Let:

- *a*_*i*_ = the expected number of new enrollments in the *i* ^th^ month (where *a*_*i*_ is constant across all *i*)
- *b*_*m*_ = the expected number of re-randomizations in month *i* = *m*
- *CR*_*m*_ = the cumulative recruitment at the end of month *i* = *m*
- *D* = the final month of recruitment (i.e. *D* = 27 if the recruitment duration for a parallel-group trial is 27 months)
- *c*_*i*_ = for patients who were newly enrolled in month *i*, *c*_*i*_ represents the expected number of treatment episodes they will experience during months *i + 2* to *D*. (Note that we used *i + 2* to exclude exacerbations (exacerbations) that occurred during the 1-month follow-up period from the patient’s initial enrollment)

Then, for a limit of two enrollments per participant, we calculated *c*_*i*_ as:

$$ {c}_i={a}_iP\left(X\ge 1\right), $$

where *X* denotes the number of episodes that occur during months *i + 2* to *D* for any particular patient. We calculated *P*(*X* = *x*) based on a Poisson distribution with a rate 0.087(*D* − *i* − 1).

For a limit of four enrollments per participant, we calculated *c*_*i*_ as:

$$ {c}_i={a}_iP\left(X=1\right)+2{a}_iP\left(X=2\right)+3{a}_iP\left(X\ge 3\right). $$

We assumed that exacerbations (*c*_*i*_) occurred uniformly throughout the remaining recruitment period. We then calculated the expected number of re-randomizations in month *m* (*b*_*m*_) as:

$$ {b}_m={\displaystyle \sum_{i=1}^{m-1}}\frac{c_i}{D-i-1}. $$

We then calculated the expected cumulative recruitment (*CR*) at month *m* as:

$$ C{R}_m={\displaystyle \sum_{i=1}^m}\left({a}_i+{b}_i\right). $$

## Abbreviations

IVF: In vitro fertilization
RCT: Randomized controlled trial
